# Suppression of experimental uveitis by a recombinant adeno-associated virus vector encoding interleukin-1 receptor antagonist

**Published:** 2009-08-08

**Authors:** Ming-Ling Tsai, Chi-Ting Horng, Show-Li Chen, Xiao Xiao, Chih-Hung Wang, Yeou-Ping Tsao

**Affiliations:** 1 Department of Ophthalmology, Tri-Service General Hospital, Taipei, Taiwan; 2 Department of Ophthalmology, Mackay Memorial Hospital, Taipei, Taiwan; 3 Department of Molecular Genetics and Biochemistry, University of Pittsburgh School of Medicine, Pittsburgh, PA; 4 Department of Microbiology and Immunology, National Taiwan University, Taipei, Taiwan; 5 Department of Ophthalmology, Kaohsiung Armed Force General Hospital, Kaohsiung, Taiwan; 6 Department of Pharmacy, Tajen University, Pintung, Taiwan; 7 Department of Otolaryngology-Head and Neck Surgery, Tri-Service General Hospital, Taipei, Taiwan; 8 Department of Ophthalmology, Taichung Veterans General Hospital, Taichung, Taiwan; 9 Department of Ophthalmology, National Defense Medical Center, Taipei, Taiwan

## Abstract

**Purpose:**

To evaluate the potential of gene therapy with a recombinant adeno-associated virus vector encoding the interleukin-1 receptor antagonist gene (rAAV-IL-1Ra) in the treatment of experimental uveitis.

**Methods:**

The vitreal cavity of New Zealand white rabbits was injected with rAAV-IL-1Ra (4×10^7^ infectious units), and the contralateral eye was injected with the same amount of rAAV-LacZ or PBS as a control. Transgene expression was evaluated by immunohistochemistry, ELISA, and RT-PCR. To evaluate the therapeutic potential of rAAV-IL-1Ra, experimental uveitis was induced by intravitreal injection of IL-1α at 10 and 100 days after rAAV–IL-1Ra administration. The effects of rAAV-IL-1Ra on experimental uveitis were investigated using histological and aqueous analysis.

**Results:**

Following intravitreal injection of rAAV-IL-1Ra, transgene expression was found in various cell types of the ocular tissues, such as ciliary epithelial cells, retinal ganglion cells, and retinal pigment epithelial cells. RT-PCR and ELISA showed that the IL-1Ra transgene persisted in the rabbit eye for at least 100 days. Compared with the control eyes, the transgene expression ameliorated experimental uveitis at 10 and 100 days after a single administration of rAAV-IL-1Ra.

**Conclusions:**

Intravitreal administration of rAAV-IL-1Ra led to sustained human IL-1Ra transgene expression in rabbit eyes for 100 days. The transgene expression suppressed uveitis episodes at 10 and 100 days after rAAV-IL-1Ra injection. Long-term suppression of experimental uveitis could be achieved by gene therapy with rAAV-IL-1Ra.

## Introduction

Uveitis is the most common form of intraocular inflammation. Uveitis affects 0.2% of the population and has an annual incidence of 8.2 new cases per 100,000 people [1]. The process of uveitis is refractory and recurrent [2]. The duration of chronic uveitis can exceed three months and that of acute anterior uveitis more than four weeks [2,3]. Fifty percent of uveitis patients experience recurrent episodes that may lead to serious and vision-threatening ocular complications [2,3].

Steroid therapy is the current principle treatment for uveitis [4]. However, long-term administration of steroids is complicated by many side effects, such as infection, cataracts, and glaucoma [4-6]. Therefore, the development of alternative treatments to control this intraocular inflammation is necessary.

The interleukin-1 receptor antagonist (IL-1Ra) is a member of the IL-1 family. IL-1Ra binds to the IL-1 receptor but does not induce any intracellular response [7-10]. Previous studies have reported that IL-1Ra improves many ocular inflammatory diseases, such as conjunctivitis and corneal graft rejection [11,12]. Experimental uveitis can also be suppressed by administration of IL-1Ra protein [13,14]. However, as the half-life of IL-1Ra protein is short (15–25 min), it is thus impractical for clinical uveitis therapy [15].

Advances in molecular biology suggest that gene therapy with the IL-1Ra-encoding gene has potential for the treatment of uveitis. If the IL-1Ra-encoding gene can be delivered into the target cells and this results in the stable expression of that gene, such a strategy may overcome the problem of the short half-life of functional IL-1Ra protein. To date, naked DNA-, retroviral-, and adenovirus-based vectors have been studied as potential agents in ocular gene therapy [16,17]. However, the studies mentioned above indicate that these vectors have some limitations in achieving ideal ocular gene therapy, including poor delivery efficiency, lack of sustained expression, and host immune reactions [17-19]. The adeno-associated virus (AAV) is a promising gene delivery system because this vector might be used to deliver appropriate genes into diverse cell types in many tissues without causing significant host immune reactions [18,20,21]. There are reports of recombinant AAV (rAAV) vectors that can deliver a transgene into the cells of various tissues, such as the central nervous system, muscle, lung, and gut, and produce long-term expression [18,20,21]. In our previous studies, we found that an rAAV vector can efficiently deliver the transgene into various cells in joint and ocular tissues and produce long-term expression, and that experimental arthritis can be suppressed and prevented by gene therapy with rAAV-IL-1Ra [22,23]. These findings have led us to further explore the potential of using an rAAV vector to deliver the IL-1Ra-encoding gene for uveitis gene therapy.

In this study, we evaluated the effects of gene therapy with rAAV-IL-1Ra on experimental uveitis. The rAAV vector encoding the human IL-1Ra-encoding gene, which is driven by the human cytomegalovirus (CMV) promoter, was introduced into the eyes of rabbits by intravitreal injection. The expression of the rAAV-mediated transgene in ocular tissue and the effects of rAAV-IL-1Ra on experimental uveitis were evaluated.

## Methods

### Animals and experimental uveitis

New Zealand white rabbits (weighing 2–3 kg) were handled in accordance with the ARVO guidelines. The rabbits were anesthetized by intramuscular injection containing a mixture of 35–50 mg/kg ketamin and 8–12 mg/kg xylazine. Experimental uveitis was induced by interleukin-1 (IL-1) α [14,24]. In summary, human IL-1 α (Sigma, St. Louis, MO) was dissolved in distilled water with gentle sonication and diluted to 0.5 ng/μl in PBS. Ten nanograms (ng) of IL-1 alpha was injected into the vitreal cavity of the rabbit eye through the pars plana using a 30 gauge needle. After injection, gentamicin ointment was applied.

### Vector construction and rAAV production

Human IL-1Ra complementary DNA (cDNA) was obtained by reverse transcription-polymerase chain reaction (RT-PCR) from RNA prepared from U937 cells that were treated with phorbol ester for 48 h [10]. The forward primer used in the PCR reaction was 5′-TA gcg gcc gc ATG GAA ATC TGC-3′, which not only spans the regions converting the initiation codon (underlined) and its flanking sequences but also contains a Not I site (the lowercase letter) at the 5′ end. The reverse primer was 5′-AA gcg gcc gc CTA CTC GTC CCT C-3′, which not only spans the stop codon (underlined) and its flanking sequences but also contains a Not I site (the lowercase letter) at the 5′ end [22]. The IL-1Ra gene was cloned between the two Not I sites of pXX-UF1 to replace the green fluorescent protein gene, and was thus placed under the transcription regulation of the human cytomegalovirus (CMV) immediate-early promoter. Recombinant AAV encoding human IL-1Ra cDNA was constructed using a three-plasmid cotransfection system, as previously described in the literature [22,23,25-27]. Titers of rAAV-LacZ and rAAV-IL-1Ra were determined by dot-blot hybridization.

### Histochemical detection of β-galactosidase

Rabbit eyes were enucleated and prefixed by immersion in 4% paraformaldehyde-PBS (pH 7.4) on ice for 15 min. The eyeballs were rinsed twice in PBS and then incubated in a dark room for 12 h at 37 °C with 5-bromo-4-chloro-3-indolyl-β-d-galactosidase (X-gal; Calbiochem, La Jolla, CA) in a solution containing 10 mM K_3_Fe(CN)_6_, 10 mM K_4_Fe(CN)_6_, 2 mM MgCl_2_, 0.01% deoxycholate, and 0.02% NP40 in PBS (pH 7.8). The eyes were postfixed for 6 h in 2% glutaraldehyde and 4% formaldehyde-PBS (pH 7.0), cryoprotected by sequential soaking in 10% and 30% sucrose solution, placed in optimal cutting temperature (OCT) compound (Miles Laboratories, Elkhart, IN), and then snap frozen in liquid nitrogen and cut into 8 μm sections. The sections were counterstained with eosin and examined for the *E. coli* β-galactosidase (LacZ) gene signal.

### Immunohistochemistry assay

The rabbits were euthanized by intracardial injection with pentobarbital, and the eyes removed and quick-frozen in OCT embedding compound (Miles Laboratories). Ten-micrometer sections were cut, mounted on microscope slides, and then air-dried for 30 min. After fixation in 4% paraformaldehyde for 1 h, sections were rinsed in PBS and the endogenous peroxidase was then quenched with 1.0% H_2_O_2_ for 5 min at room temperature. The sections were rinsed twice in PBS, and then blocked with a mixture of 5% bovine serum albumin and 0.3% Triton X-100 in PBS for 30 min. A goat polyclonal antibody to human IL-1Ra (R&D Systems, Minneapolis, MN) was added to the sections for 1 h. Primary antibodies were localized by immunoperoxidase staining (R&D Systems). The sections were then washed three times with PBS, followed by the addition of biotinylated anti-goat secondary antibodies for 1 h. After rinsing in PBS, the samples were incubated with streptavidin-peroxidase for 30 min. After washing, the sections were reacted with the DAB (3,3-diaminobenzidine tetrahydrochloride) chromogen. The sections were rinsed with distilled water and counterstained with hematoxylin. They were then mounted on microscope slides using a xylene-based mounting medium, and observed under a microscope.

### Reverse transcription-polymerase chain reaction (RT-PCR)

The eyeballs were enucleated and frozen immediately in liquid nitrogen. The samples were homogenized in guanidine thiocyanate, and total RNA was extracted by phenol-chloroform. One microgram of the RNA was used for reverse transcription. Complementary DNA was synthesized using oligo(dT) as a primer and 200 IU of SuperScript II transcriptase (Gibco BRL, Gaithersburg, MD) according to the manufacturer’s instructions. PCR amplification was done as described above for vector construction. The housekeeping gene, Βeta-actin, served as a control to ensure that equal amounts of RNA were analyzed from each sample. The upstream primer sequence for β-actin was 5′-AGG CCA ACC GCG AGA AGA TGA CC-3′, and the reverse primer was 5′-GAA GTC CAG GGC GAC GTA GCA C-3′, which was expected to produce a 350 bp DNA fragment [25]. The PCR products were separated by 1% agarose gel electrophoresis, stained with ethidium bromide, and photographed.

### Enzyme-linked immunosorbent assay (ELISA)

The rabbit eyes were enucleated and uveoretinal tissues such as the iris, ciliary body, retina, and choroid-sclera were harvested. The collected tissues were homogenized in 10–20 volumes of homogenization buffer (50 mM Tris, 0.3MNaCl, and 0.3% Triton X-100) containing protease inhibitors (0.1 mM phenylmethylsulfonyl fluoride, 0.1 mM benzethonium chloride, 1 mM benzamidine, 1 mM ethylenediaminetetraacetic acid, and 200 KIU/ml aprotinin). The homogenates were centrifuged at 14,000x g for 30 min. The supernatants were collected and total protein concentrations were detected using the Bio-Rad protein assay system (Bio-Rad Laboratories, Hercules, CA). Human interleukin-1Ra levels were determined with a sandwich enzyme-linked immunosorbent assay, according to the manufacturer’s instructions (R&D Systems) [27,28].

### Aqueous cell counting and protein concentration

Rabbits were prepared and anesthetized as usual. The aqueous humor was aspirated by puncturing the anterior chamber with a 30 gauge needle, and the fluid loaded onto a hemocytometer for aqueous cell counting [14,29]. To determine protein concentration, the aqueous humor was centrifuged at 3,000 rpm for 5 min to remove cell debris. The supernatant was collected, and the protein concentration determined by the Lowry method [14,30].

### HE staining

The eyes were enucleated and fixed in 4% paraformaldehyde for 24 h. The fixed tissue was embedded in paraffin. Ten μm sections were cut and mounted on microscope slides, and the sections were stained with hematoxylin and eosin. These slides were examined and photographed under a microscope.

### Statistical analysis

One-way ANOVA tests were used to analyze the differences between experimental and control groups (rAAV-LacZ and PBS) in each assay. All data were expressed as mean ±standard deviation. If the overall F-test was significant, Tukey post-hoc comparisons were used to detect significant differences among different groups. The p-value < 0.01 was considered significant.

## Results

### Experimental uveitis

**Figure 1:**
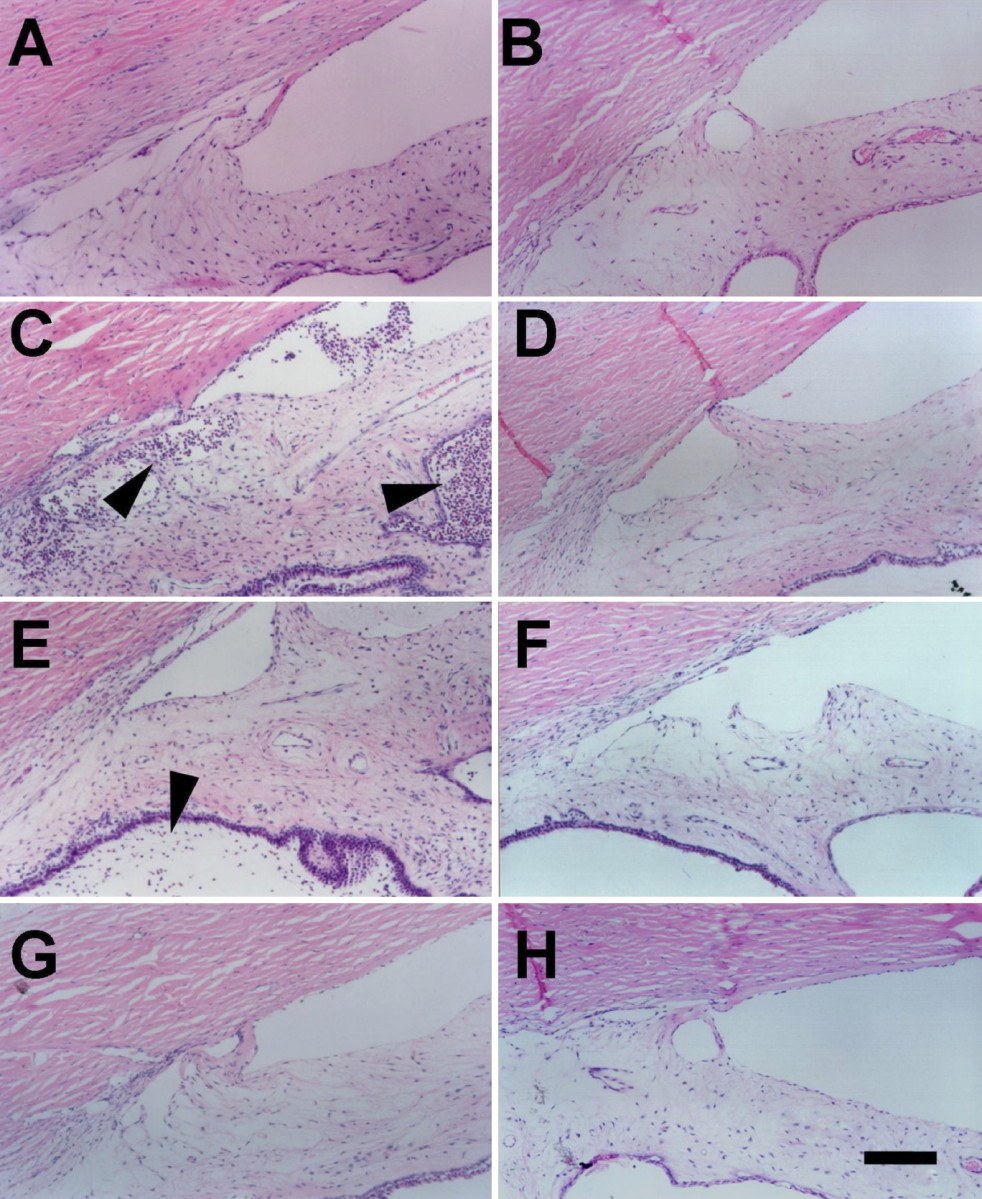
Establishment of experimental uveitis. The vitreal cavity of rabbit eyes was injected with IL-1α to induce experimental uveitis, and the contralateral eye was injected with the same amount of the vehicle (distilled water) as a control. Inflammation was evaluated by the presence of leukocyte infiltration (arrowheads) in the tissues of the anterior ocular segment. Immediately before IL-1α or vehicle injection, no leukocyte infiltration was observed (**A**, **B**). The inflammation, defined as the presence of massive leukocyte infiltration in the tissue of the anterior ocular segment, peaked one day after IL-1α injection (**C**). Three days after IL-1α injection, the inflammation had partially subsided (**E**), and 15 days after injection, no sign of inflammation was seen in the anterior ocular segment (**G**). Experimental uveitis was not detected in the control eyes at 1, 5, or 15 days after vehicle injection in the control group (**B**, **D**, **F**, **H**). The scale bar in **H** is equal to 100 μm.

To study the potential effects of rAAV-IL-1Ra on uveitis, we needed to establish an animal model of experimental uveitis. Experimental uveitis was induced by intravitreal injection of IL-1α (10 ng) in New Zealand white rabbits (Figure 1C,E,G). The contralateral eye was injected with the same amount of the vehicle (distilled water; Figure 1D,F,H) to serve as a control [14,24,30]. Inflammation was evaluated by the presence of leukocyte infiltration. Before IL-1α or vehicle injection, no signs of inflammation were found in the ocular tissues (Figure 1A,B). Following IL-1α injection, profound leukocyte infiltration and protein accumulation were observed in the tissues of the anterior ocular segment. The inflammation was noted within 6 h, peaked at 24 h (Figure 1C, arrowhead), and decreased thereafter. Three days after IL-1α injection, moderate inflammation was seen (Figure 1E), and at 15 days after injection, no inflammation was identified in the anterior ocular segment (Figure 1G). Experimental uveitis was not detected in the control eyes at 1, 5, and 15 days after vehicle injection (Figure 1B,D,F,H, respectively). The histological findings demonstrated that experimental uveitis could be induced by intravitreal injection of IL-1α and that the uveitis subsided within 15 days.

### Transgene expression in rabbit eyes

**Figure 2:**
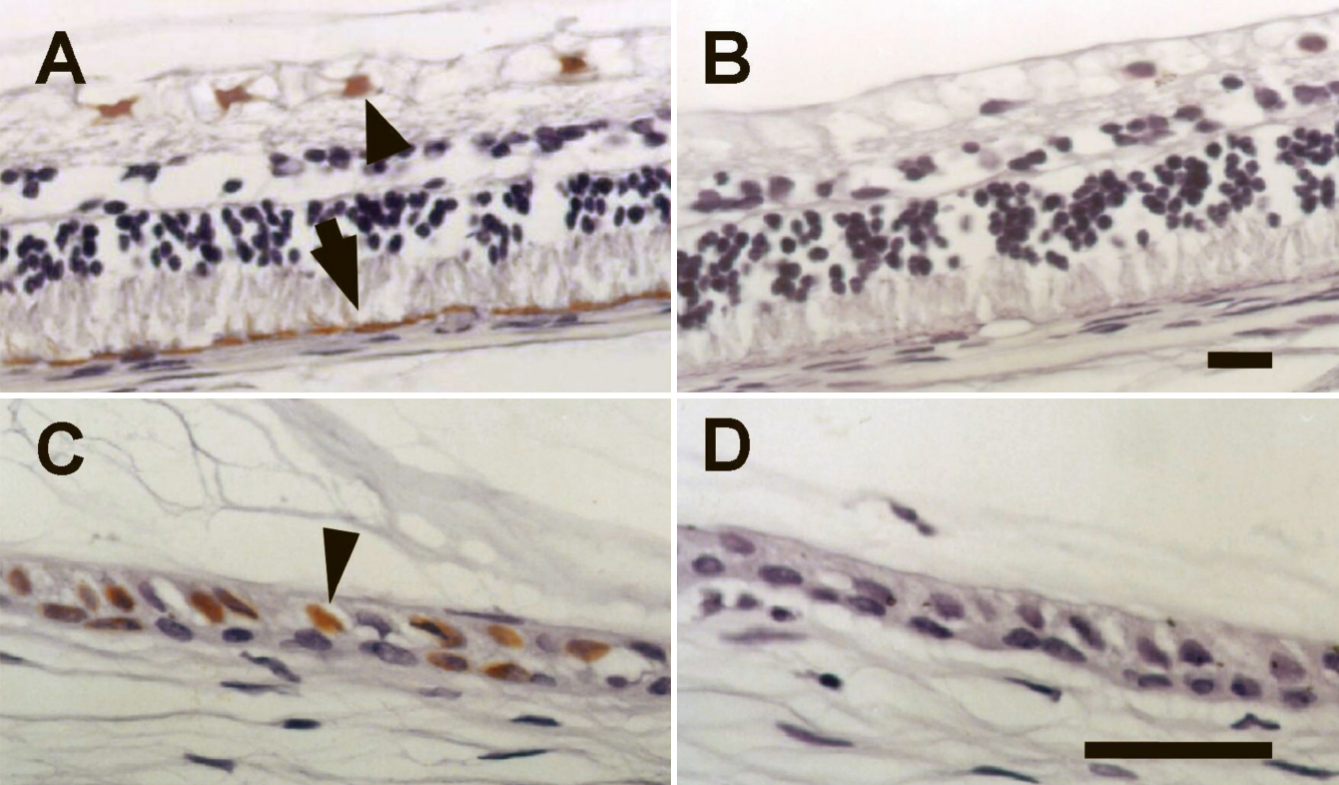
rAAV-mediated human IL-1Ra transgene expression in rabbit eyes. The vitreal cavity of rabbit eyes was injected with rAAV-IL-1Ra (4×10^7^ infectious units), and the contralateral eye was injected with the same amount of rAAV-LacZ or PBS as a control. Ten days later, the rabbit eyes were removed and processed for immunohistochemistry. In rAAV-IL-1Ra-injected eyes, transgene expression was found in various ocular tissue cell types, including retinal ganglion cells (**A**, arrowhead), retinal pigment epithelial cells (**A**, arrow), and ciliary epithelial cells (**C**, arrowhead). No human IL-1Ra transgene expression was detected in the control eyes (**B** and **D**). The scale bar in **D** is equal to 25 μm.

To localize the rAAV-mediated transgene expression, the expression of the IL-1Ra transgene was detected immunohistochemically in the ocular tissue of rabbit eyes. Brown staining was detected in the rAAV-IL-1Ra-injected eyes. IL-1Ra expression was observed as brown staining in various cell types in the uveoretinal tissues, including retinal ganglion cells (Figure 2A, arrowhead) and ciliary epithelial cells (Figure 2C, arrowhead) in all 10 rabbits. Retinal pigment epithelial (RPE) cells located in the peripheral retina also stained positively in two of the 10 rabbits’ eyes (Figure 2A, arrow). However, no positive staining was observed in the control eyes (Figure 2B,D).

**Figure 3:**
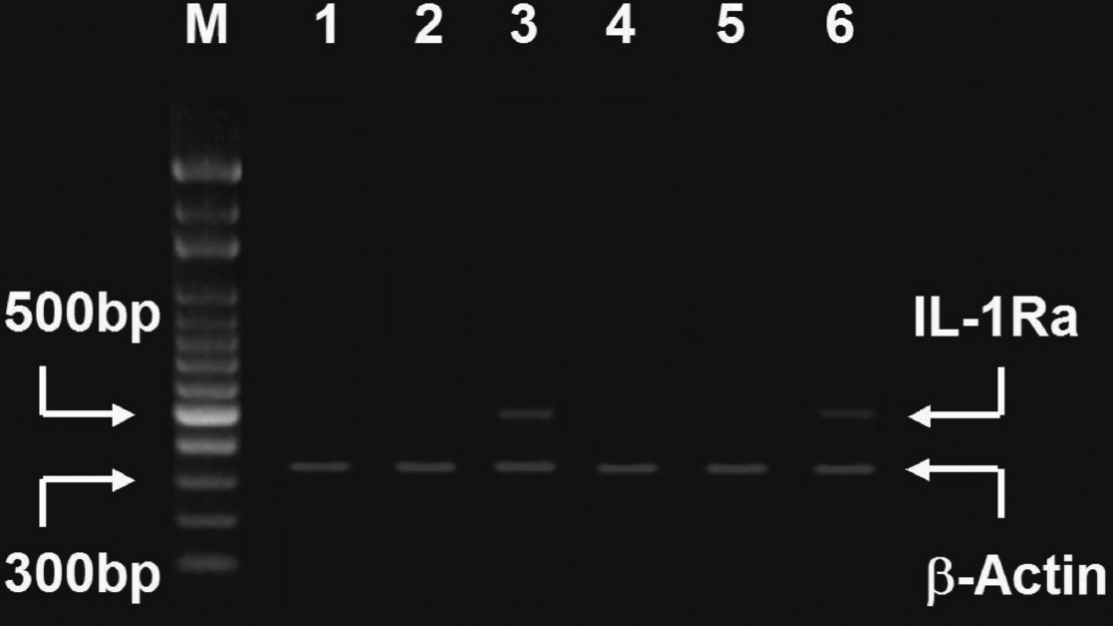
RT-PCR analysis. The vitreal cavity of rabbit eyes was injected with rAAV-IL-1Ra (4×10^7^ infectious units), and the contralateral eye was injected with the same amount of rAAV-LacZ. In another group, the right eye was injected with the same amount of PBS. Ten days later, the rabbit eyes were enucleated, extracted, and subjected to RT-PCR. No human IL-1Ra mRNA was observed in the rAAV-LacZ-injected (lane 1) or PBS-injected (lane 2) eyes. Significant transgene expression was observed in rAAV-IL-1Ra-injected eyes (lane 3). After 100 days, the eyes of different rabbits were enucleated, extracted, and subjected to RT-PCR. No human IL-1Ra mRNA was found in the rAAV-LacZ-injected (lane 4) or PBS-injected (lane 5) eyes. Significant transgene expression was still detected in the rAAV-IL-1Ra-injected eye (lane 6). Lane M shows the molecular weight markers.

To further evaluate the expression of the rAAV-mediated transgene in the rabbit eye, IL-1Ra transgene expression was followed for 100 days. RT-PCR was used to detect mRNA expression of the IL-1Ra transgene in the rabbit eyes. Intravitreal injection with rAAV-IL-1Ra (4×10^7^ infectious units; n=5) was performed in the right eye, and the same amount of rAAV-LacZ (n=5) was injected into the left eye. In another group, the right eye was injected with the same amount of PBS (n=5). At 10 days after rAAV-IL-1Ra injection, significant IL-1Ra transgene expression was seen in the rAAV-IL-1Ra-injected eyes (Figure 3, lane 3). In contrast, no IL-1Ra gene expression was observed in rAAV-LacZ-injected eyes (Figure 3, lane 1) or PBS-injected eyes (Figure 3, lane 2). To further evaluate whether the rAAV-mediated transgene was expressed for a long time in rabbit eyes, the right eyes were treated with intravitreal injection of rAAV-IL-1Ra (4×10^7^ infectious units; n=5), and the left eyes were treated with the same amount of rAAV-LacZ (n=5). In another group, the right eye was injected with the same amount of PBS (n=5). At 100 days after rAAV-IL-1Ra injection, the rabbit eyes were isolated for RT-PCR. In the control group, no transgene expression was detected in rAAV-LacZ-injected eyes (Figure 3, lane 4) or PBS-injected eyes (Figure 3, lane 5). In contrast, mRNA of IL-1Ra was still detected in rAAV-IL-1Ra-injected eyes (Figure 3, lane 6). To quantify the expression of the rAAV-mediated transgene in the rabbit eyes, the IL-1Ra protein was measured at 10 and 100 days after rAAV-IL-1Ra injection (Table 1). A high concentration of human IL-1Ra protein was found in the eyes at 10 days (67.86±3.91 pg/g; n=5) and at 100 days (63.17±4.13 pg/g; n=5) after rAAV-IL-1Ra injection. No significant IL-1Ra protein (<1.0 pg/g) was detected in the eyes injected with rAAV-LacZ or PBS at 10 or 100 days after intravitreal injection. This finding suggested that the rAAV vector delivered the human IL-1Ra gene into the rabbit eye and resulted in functional IL-1Ra protein expression.

**TABLE 1:** ELISA identification of human IL-1RA in the rabbit eye after rAAV-IL-1RA injection.

**Concentration**	**Groups**		
	**rAAV-IL-1-Ra**	**rAAV-LacZ**	**PBS**
IL-1-Ra (pg/gm) 10 days	67.86±3.91*	< 0.1	< 0.1
IL-1-Ra (pg/gm) 100 days	63.17±4.13*	< 0.1	< 0.1

rAAV-IL-1RA was injected intravitreally into the right eyes of rabbits, and the left eyes were treated with the same amount of rAAV-LacZ or PBS. At 10 and 100 days after rAAV transduction, the entire uveoretinal tissue of each eye was harvested and processed for ELISA. The asterisk indicates statistically significant differences.

### Gene therapy with rAAV–IL-1Ra ameliorated induced experimental uveitis at 10 days after rAAV–IL-1Ra administration

**Figure 4:**
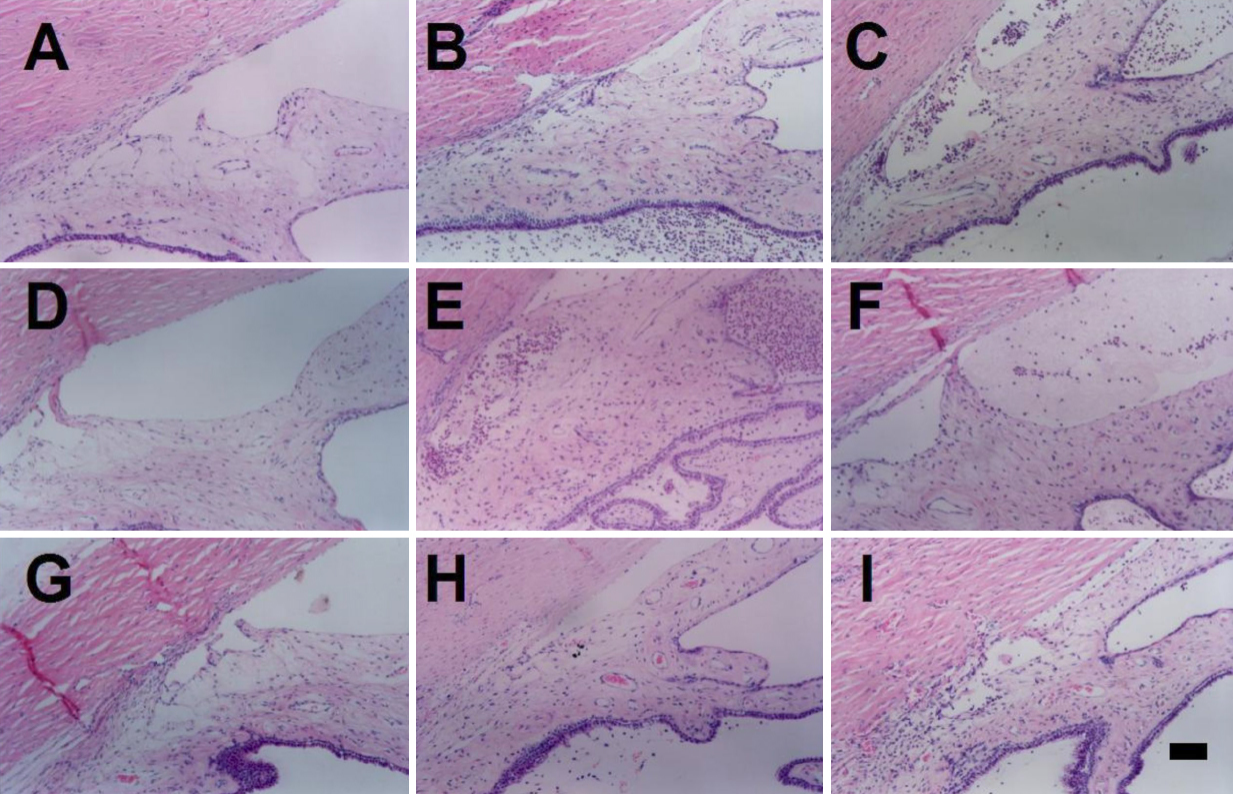
Histological findings in the rabbit eye with induced uveitis at 10 days after rAAV-IL-1Ra administration. The vitreal cavity of rabbit eyes was injected with rAAV-IL1-Ra, and the contralateral eye was injected with the same amount of rAAV-LacZ. In another group, the right eye was injected with the same amount of PBS. After 10 days, uveitis was induced by intravitreal injection of IL-1α. The eyeballs were enucleated, fixed, and stained with H & E immediately before, or one or three days after, uveitis induction. In the eyes injected with rAAV-LacZ, the time points are immediately before (**A**), one day after (**B**), and three days after (**C**) uveitis induction. In the eyes injected with PBS, the time points are immediately before (**D**), one day after (**E**) and three days after (**F**) uveitis induction. In the eyes injected with rAAV-IL-1Ra, the time points are immediately before (**G**), one day after (**H**), and three days after (**I**) IL-1α injection. The scale bar in **I** is equal to 100 μm.

**Figure 5:**
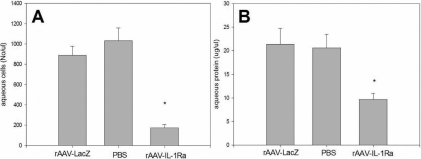
Aqueous humor analysis in the rabbit eye with induced uveitis at 10 days after rAAV-IL-1Ra administration. The vitreal cavity of rabbit eyes was injected with rAAV-IL1-Ra, and the contralateral eye was injected with the same amount of rAAV-LacZ. In another group, the right eye was injected with the same amount of PBS. After 10 days, uveitis was induced by intravitreal injection of IL-1α. At 24 hours after uveitis induction, the aqueous fluid was aspirated for analysis. The effect of rAAV-IL-1Ra was evaluated by comparing aqueous cell counts (**A**) and protein concentration (**B**) in the experimental and control groups. Values represent the mean ±standard deviation. The asterisk indicates statistically significant differences.

The effects of rAAVIL-1Ra on experimental uveitis were examined in 16 rabbits. Ten were given intravitreal injection of AAV-IL-1Ra (4×10^7^ infectious units) in the right eye, and the left eye was injected with rAAV-LacZ. In another six rabbits, the same amount of PBS was injected into the right eye. Ten days later, experimental uveitis was induced by intravitreal injection of IL-1α. At 24 h after uveitis induction, the effect of rAAV-IL-1Ra on experimental uveitis was investigated by histological examination (Figure 4) and aqueous analysis (Figure 5). In the control eyes, histological examination showed massive leukocyte infiltration and exudate accumulation in the tissues of the anterior ocular segment of rAAV-LacZ-injected eye (Figure 3B) and PBS-injected eyes (Figure 3E). Aqueous analysis was used to further investigate the effect of rAAV-IL-1Ra on the development of experimental uveitis. The aqueous cell counts were 887±91 cells/μl in rAAV-LacZ-injected eyes and 1,032±127 cells/μl in PBS-injected eyes (Figure 5A). The protein concentration was 21.4±3.3 μg/μl in rAAV-LacZ-injected eyes and 20.6±2.9 μg/μl in PBS-injected eyes (Figure 5B). The eyes treated with rAAV-IL-1Ra showed greatly attenuated inflammation. The histological examination showed minimal leukocyte infiltration and exudate accumulation in the eyes injected with rAAV-IL-1Ra (Figure 4H), and the rAAV-IL-1Ra-treated eyes had a low aqueous cell count of 173± 2 cells/μl; p <0.01 (Figure 5A) and a protein concentration of 9.7±1.3 μg/μl; p <0.01 (Figure 5B). This finding indicated that rAAV delivered the IL-1Ra transgene into the rabbit eyes and that the transgene expression suppressed experimental uveitis 10 days after rAAV-IL-1Ra injection.

### Gene therapy with rAAV-IL-1Ra suppressed induced experimental uveitis at 100 days after rAAV–IL-1Ra administration

**Figure 6:**
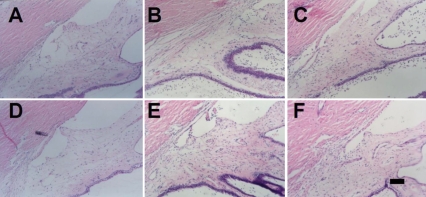
Histological findings in the eye with induced uveitis at 100 days after rAAV-IL-1Ra administration. The vitreal cavity of rabbit eyes was injected with rAAV-IL1-Ra, and the contralateral eye was injected with the same amount of rAAV-LacZ. In another group, the right eye was injected with the same amount of PBS. After 100 days later, uveitis was induced by intravitreal injection of IL-1α. Immediately before, and one or three days after, uveitis induction, the eyeballs were enucleated, fixed, and stained with H&E. Histology of eyes injected with rAAV-LacZ immediately before (**A**), one day after (**B**), and three days after (**C**) IL-1α injection. Histology of the eyes injected with rAAV-IL-1Ra immediately before (**D**), one day after (**E**), and three days after (**F**) IL-1α injection. The scale bar in **F** is equal to 100 μm.

**Figure 7:**
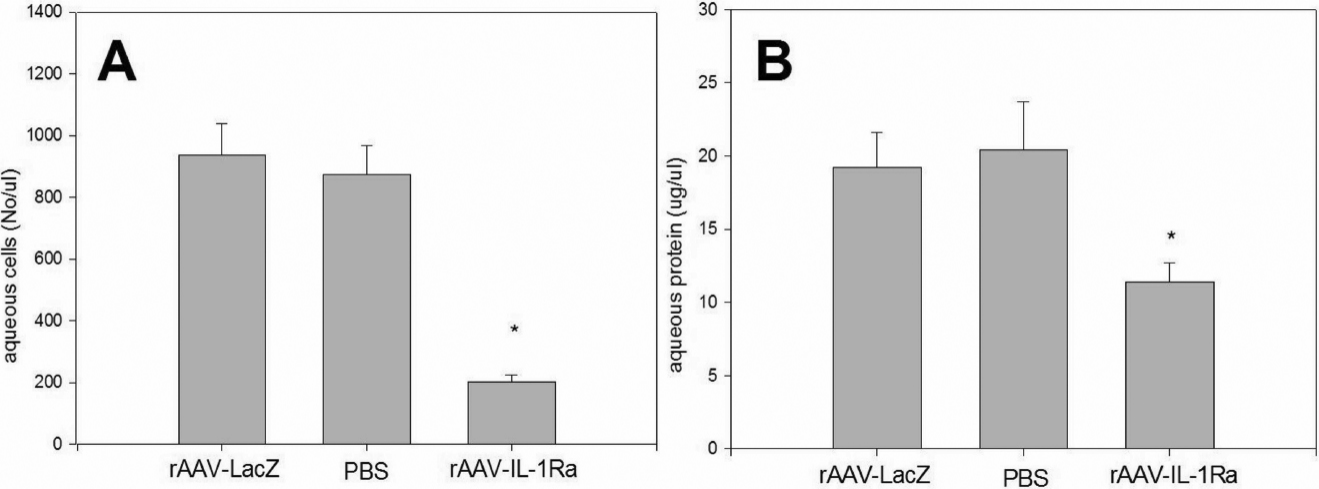
Aqueous humor analysis in the rabbit eye with induced uveitis at 100 days after rAAV–IL-1Ra administration. The vitreal cavity of rabbit eyes was injected with rAAV-IL1-Ra, and the contralateral eye was injected with the same amount of rAAV-LacZ. In another group, the right eye was injected with the same amount of PBS. After 100 days, uveitis was induced by intravitreal injection of IL-1α. At 24 h after IL-1α injection, the aqueous fluid was aspirated for analysis. The effect of rAAV-IL-1Ra on rabbit eyes with experimental uveitis was evaluated by aqueous cell counts (**A**) and protein concentration (**B**) and compared between the experimental and control groups. Values are expressed as the mean ±standard deviation. The asterisk indicates statistically significant differences.

The effects of rAAV-IL-1Ra on experimental uveitis were examined in 16 rabbits. Ten were given intravitreal injection of rAAV-IL-1Ra (4×10^7^ infectious units) in the right eye, and the left eye was injected with rAAV-LacZ. In another six rabbits, the same amount of PBS was injected in the right eye. After 100 days, experimental uveitis was induced by an intravitreal injection of IL-1α in the eye. At 24 h after uveitis induction, the therapeutic effect of IL-1Ra on experimental uveitis was evaluated by histological examination (Figure 6) and aqueous analysis (Figure 7). In the control eyes, histological examination showed massive leukocyte infiltration and exudate accumulation in the tissues of the anterior ocular segment (Figure 6B). Aqueous analysis was used to investigate the effects of rAAV-IL-1Ra on this uveitis episode. The aqueous cell count was 936±103 cells/μl in rAAV-LacZ-injected eyes and 874±93 cells/μl in PBS-treated eyes (Figure 7A). Protein concentration was 19.2±2.4 μg/μl in rAAV-LacZ-injected eyes and 20.4±3.3 μg/μl in PBS-injected eyes (Figure 7B). The eyes treated with rAAV–IL-1Ra showed greatly attenuated inflammation. Pathological examination showed minimal leukocyte infiltration and exudate accumulation in the eyes injected with rAAV-IL-1Ra (Figure 6E). The aqueous fluid from the eyes treated with rAAV-IL-1Ra had low cell counts of 203±21 cells/μl; p <0.01 (Figure 7A) and protein concentrations of 11.4±1.3 μg/μl; p <0.01 (Figure 7B). Our study showed that IL-1Ra transgene expression retained the ability to suppress experimental uveitis at 100 days after rAAV-IL-1Ra administration.

## Discussion

Uveitis is the most common intraocular inflammation. It results from disruption of the blood–ocular barrier and is characterized by leukocyte infiltration and protein leakage [1,7,31]. Although the etiology and pathogenesis of uveitis are not well elucidated, there are several animal models for understanding disease pathogenesis and testing new therapies for ocular inflammatory diseases, such as IL-1 induced uveitis, experimental autoimmune uveitis (EAU), endotoxin-induced uveitis (EIU), etc [24,30,32,33]. Among these models, IL-1 induced experimental uveitis is considered to be an animal model for acute anterior ocular inflammation. This inflammation is characterized by protein leakage in the anterior chamber and by infiltration of macrophages and neutrophils into the eye, with a peak at 24 h after IL-1 injection [24,32]. Past studies have also revealed that IL-1 may activate the T cell-mediated immune response and thereby play an important role in the pathogenesis of recurrent uveitis [34,35]. Furthermore, previous investigations have reported that IL-1 contributed to the local inflammatory response during primary infections or during recurrent episodes of toxoplasma-induced retinochoroiditis [36]. These clinical, immunological, and histological characteristics make IL-1 induced uveitis a suitable in vivo model to gain information regarding the immunopathogenesis and treatment of inflammatory ocular diseases in humans.

In this study, we administered rAAV-IL-1Ra to the peripheral retina and found that the rAAV vector efficiently delivered the transgene to the ganglion cell and ciliary epithelial cells in all rAAV-IL-1Ra-injected eyes. Efficiency is critical for successful ocular gene therapy. In this study, the AAV vector used was the AAV2 serotype, because this vector can transfer the gene into various ocular cells through intravitreal or subretinal injection. Over 100 different AAV serotypes have been described, several of which have already been tested in vivo in animals [37-39]. In ocular gene therapy, AAV vectors based on serotypes 1, 2, 4, and 5 have been evaluated after intravitreal and subretinal injection [37,39]. Of these, AAV2 is considered the only vector able to efficiently transduce cells into the inner retinal layers after intravitreal injection [27,37,39,40]. Previous research has reported that intravitreal injection of AAV2 led to the transduction of retinal ganglion cells and various cells of the inner nuclear layer for months [27,29,40]. Past studies have also revealed that subretinal injection of the AAV2 vector delivering a reporter gene results in transduction of RPE and photoreceptor cells in adult mice [26,37,39]. On the other hand, AAV1, 4, and 5 vectors delivered transgenes efficiently to the photoreceptor and pigment epithelium after subretinal injection. However, little transgene expression is observed following intravitreal injection of these vectors [37,39]. Therefore, the AAV2 vector was chosen to be tested for potential uveitis gene therapy. In this study, we also found transgene expression in RPE cells in two of the 12 injected eyes. This transgene expression in RPE cells was found in the retina adjacent to the injected site. We assume that the rAAV vector may penetrate or infiltrate into the subretinal space at the junction of the pars plana and peripheral retina, because the retina around this area is relatively thin and attached loosely to choroid.

Our results also indicate that experimental uveitis induced by IL-1 at 10 and 100 days after rAAV-IL-1Ra administration could be suppressed by a single intravitreal injection of rAAV-IL-1Ra. Previous studies have reported that IL-1Ra is a potential anti-inflammatory agent in the treatment of uveitis [14,30,41]. The IL-1Ra is an endogenous peptide that binds to IL-1 receptors but does not induce any intracellular response [8]. IL-1Ra may suppress experimental uveitis by protecting the blood–ocular barrier from disruption by IL-1 stimulation [14,30]. Because the amino acid sequence of human IL-1Ra shows 77% homology to that of rabbit IL-1Ra, Rosenbaum et al. [14] reported that intravitreal injection with functional human IL-1Ra protein successfully inhibits experimental uveitis in rabbits. Moreover, the functional human IL-1Ra protein expression in the cells transduced by the rAAV vector in this study might be effective in protecting the blood–ocular barrier from IL-1 insult. Previous research has also shown that these transduced cells are important for maintaining the blood–ocular barrier, which includes the zonula occludens between the ciliary epithelial cells, tight junctions between retinal vessel endothelial cells, and tight junctions between RPE cells [31]. Therefore, we observed that experimental uveitis was ameliorated at 10 days after rAAV-IL-1Ra injection. Moreover, the observation that rAAV-mediated transgene expression persisted for at least 100 days also explains that induced experimental uveitis episodes can be suppressed at 100 days after rAAV-IL-1Ra administration.

Although gene therapy with rAAV vectors is a promising approach in the treatment of experimental uveitis, there are still several improvements required to achieve practical management in the treatment of clinical uveitis in humans. Previous research has revealed that transgene expression decreases over time because most rAAV-mediated transgenes are episomal, and only a minority integrate randomly into the host genome [42,43]. Moreover, intravitreal administration of AAV vectors can elicit neutralizing antibodies against the vector capsid, thus decreasing the efficiency of therapeutic gene transfer and preventing effective vector readministration [44]. Thus, the stability of rAAV-mediated transgene expression remains a challenge. Furthermore, long-term constitutive transgene expression is not always desirable under certain conditions. In diseases such as intraocular inflammation, the level and timing of transgene expression must be regulated [23,25]. Gene therapy with cell-specific and inducible expression systems may offer an attractive strategy for obtaining therapeutic efficacy and avoiding toxicity [22,23,25]. Additional modification of the AAV vector design might include a cell-specific and inflammation-inducible promoter system. In addition, various cell transduction and exogenous therapeutic gene products may compromise the functioning of the neuroretina [38]. There is a theoretical risk associated with gene therapy with an AAV vector, although previous studies report that the rAAV vector is a safe gene delivery system [27]. Besides, clinical uveitis can be caused not only by IL-1 but also by other cytokines such as IL-6, IL-10, and tissue necrosis factor, etc [30,45,46]. Thorough investigation of the roles of various cytokines may elucidate the pathogenesis and provide potential approaches for the treatment of uveitis. Further investigation is necessary.
